# Human papillomavirus vaccination: knowledge, acceptance, and barriers among medical students. An Egyptian multicenter cross sectional study

**DOI:** 10.1038/s41598-026-51547-2

**Published:** 2026-06-15

**Authors:** Doaa I. Omar, Salman Althobaiti, Nashwa Nabil, Shimaa G. Mohammed, Basma M. Hani

**Affiliations:** 1 Department of Community, Environmental and Occupational Medicine, Benha Faculty of Medicine, Benha University, Benha, Egypt; 2 Department of Family, Community, and Medical Education, Faculty of Medicine, Taibah University, Madinah, Saudi Arabia

**Keywords:** HPV vaccine, Knowledge, Attitude, Acceptance, Hesitancy, Willingness, Egypt, Cancer, Health care, Psychology, Psychology

## Abstract

Human papillomavirus (HPV) infection is a major public health concern and a leading cause of cervical cancer worldwide. Despite the availability of effective vaccines, awareness and vaccine acceptance remain limited in many developing countries. This study aimed to assess awareness, acceptance, barriers, and determinants of HPV vaccination among medical students in Egypt. A web-based multicentre cross-sectional study was conducted among undergraduate medical students from governmental, national, and private medical faculties across Egypt using a non-probability sampling approach (convenience and snowball sampling). Data were collected using a validated structured questionnaire. A total of 601 students participated in the study. 31.4% were unaware that HPV is available for both males and females, and half of them expressed negative attitudes and concerns regarding the novelty (44%), safety (56%), efficacy (59%), and cost of the vaccine (51.7%). Nearly half (48.3%) of them were unaware that the “HPV vaccine” is available in Egypt. Seventy-three students (12.1%) received the vaccine, and among vaccinated participants, 39 were males. In addition, 44.3% of students reported hesitancy toward HPV vaccination, while 58.9% indicated willingness to receive the vaccine if it was provided free of charge. Family history of cervical cancer, total knowledge, and attitude scores were significant predictors of participants’ HPV vaccine uptake (*p* < 0.05). Nearly half of the participants had not taken the vaccine because they were not sexually active or they lacked knowledge. Unfortunately, about one-third of the participants didn’t take the vaccine because of cultural and parental objections. Male participants were less likely to recommend the vaccine to others. In spite of being medical students, their knowledge and attitudes were less than expected, giving an idea about the public situation. Findings underscore the importance of educational campaigns to raise knowledge and change faulty beliefs, focusing on the target groups and their parents. Also, the national health authorities should allocate resources to make the HPV vaccine available, accessible, and affordable.

## Introduction

Cervical cancer is a major global public health challenge and remains one of the leading causes of cancer-related morbidity and mortality. Worldwide, an estimated 620,000 women and 70,000 men developed cancer in 2019 due to HPV^1^. It is primarily caused by persistent infection with high-risk types of human papillomavirus (HPV), particularly types 16 and 18, which account for approximately 70% of cases^2^. Despite a long latency period that may extend up to 15–20 years, cervical cancer is largely preventable through effective vaccination and screening strategies^3^.

In response to this burden, the World Health Organization (WHO) launched a global strategy in 2020 targeted at eliminating cervical cancer as a public health problem. This strategy emphasizes a comprehensive approach including HPV vaccination, screening, early detection, and appropriate treatment, with a goal to achieve 90% HPV vaccination coverage by 2030^4^.

Vaccination against HPV is the most important part of primary prevention. As of 2023, there are six HPV vaccines available around the world. These vaccines have been shown to be safe and effective at preventing HPV infection and cervical cancer. These vaccines protect against the most common high-risk HPV types, especially types 16 and 18. Girls between the ages of 9 and 14 should get vaccinated, preferably before they start having sex. Depending on the regimen, one or two doses may be given. People with weak immune systems should get two or three doses. Some countries have also expanded vaccination programs to include males in order to lower the spread of HPV and stop cancers linked to HPV^3^.

However, substantial disparities persist, particularly in low- and middle-income countries where the majority of cervical cancer deaths occur^5^. In Egypt, the HPV vaccine has not yet been incorporated into the national immunization program and is only available through self-payment, creating significant financial and accessibility barriers. Also, the lack of organized national screening programs further limits early detection and prevention efforts^6^.

Addressing these gaps, we need a strong healthcare workforce that can promote preventive strategies. In this context, medical and healthcare students constitute a vital target demographic, as they are the future healthcare providers who will significantly contribute to patient education, vaccine advocacy, and the execution of national prevention initiatives^7^. Their knowledge, attitudes, and acceptance of HPV vaccination are essential determinants in how many people will get it in the future^8^.

Previous studies conducted in Egypt have explored awareness and attitudes toward HPV vaccination among different population groups, including students and the general public, demonstrating variable levels of knowledge and acceptance^9,10^. However, most of these studies were limited to single institutions, focused on isolated aspects such as knowledge or attitudes, and did not comprehensively assess the range of perceived barriers influencing vaccine acceptance.

Furthermore, there is a lack of multi-institutional evidence that simultaneously evaluates awareness, acceptance, and specific perceived barriers to HPV vaccination among medical students in Egypt, limiting the generalizability of existing findings and their applicability to national-level interventions.

Therefore, this study aimed to assess the knowledge, acceptance, and specific perceived barriers toward HPV vaccination among medical students across multiple medical educational institutions in Egypt. As being the future health care providers and medical instructors, assessing their knowledge and attitudes toward HPV vaccines is a corner stone for improving future clinical practice and public health outcomes.

## Methods

### Study type, setting, and target population

This web-based, multicenter, analytic cross-sectional study was conducted during the period from October 2024 to January 2025. The study targeted undergraduate medical students, including interns, from 10 governmental, national, and private medical faculties across Egypt.

Participants were recruited through online platforms and official medical student groups affiliated with the participating universities. To verify eligibility, the first section of the questionnaire required participants to indicate their university name, faculty of medicine, and academic year (including internship year). Only respondents who confirmed that they were current undergraduate medical students or interns enrolled in a faculty of medicine in Egypt were included in the study (including Egyptian and non-Egyptian students). Participants who did not meet the eligibility criteria didn’t fill out the questionnaire.

All methods were performed in accordance with the relevant guidelines and regulations.

- **Inclusion criteria**:
  - Undergraduate medical students (including interns) enrolled in Egyptian medical faculties who provided informed consent to participate in the study.
- **Exclusion criteria**:
  - Participants who declined to participate in the study.
  - Participants diagnosed with cervical cancer.

### Sampling technique

A non-probability sampling approach was used, specifically convenience sampling combined with snowball sampling. The online questionnaire was initially distributed to undergraduate medical students through social media platforms and academic student groups affiliated with different faculties of medicine across Egypt (e.g., student groups on WhatsApp, Telegram, and Facebook). These groups are commonly used by medical students for academic communication.

Participants who received the survey link were encouraged to share it with their colleagues from other medical schools, thereby facilitating snowball recruitment and expanding the reach of the survey to a wider population of medical students from different universities.

### Sample size

The sample size was calculated using the single population proportion formula, based on a previously reported prevalence of unsatisfactory knowledge level (76.7%) from an Egyptian study, Ali E, 2024^11^. The sample size was estimated using Daniel, 1999 formula^12^:

N=Z^2^P(1 − P)/d^2^. Where:

- **N** = minimum required sample size.
- **Z** = standard normal variate at a 95% confidence level (1.96).
- **P** = expected proportion in the population based on previous studies (0.84).
- **d** = margin of error (precision) set at 0.05.

Accordingly, the minimum required sample size was 275. using a design effect of two to account for the non-probability sampling, the target sample size was increased to 550 participants. To enhance the statistical power of the study, efforts were made to maximize participant recruitment, resulting in a final sample of 601 participants during the study period.

### Data collection tool

After an extensive literature review, a structured and previously tested questionnaire was adopted from^13–15^.

The questionnaire comprised six sections:

- **Section 1: **An introductory part that outlined the aim of the study, the target group, and the voluntary nature of medical students’ participation.
- **Section 2: **Socio-demographic characteristics, including age, sex, residence, governorate, university, family history of cervical cancer, and GPA.
- **Sections 3 and 4: **Adapted, and pilot-tested English questionnaire on knowledge and attitudes regarding HPV, adopted from previous studies^14,15^. Section 3 assessed the HPV knowledge component, which is composed of general knowledge about HPV and its vaccine through nine items, on a three-point Likert scale (do not know = 0 to correct answer = 2). The maximum score for this part is 18, and the minimum score is zero. In addition to the assessment of knowledge of HPV and vaccination among females, on a five-point Likert scale (1 = strongly disagree to 5 = strongly agree), the maximum score for this part is 25, and the minimum is 5. The overall knowledge score is the sum of both components (the maximum score is 43, and the minimum is 5). Participants scoring ≥ the median score were considered to have a satisfactory knowledge level. Section 4 evaluated participants’ attitudes toward HPV vaccination. It included seven items addressing female HPV vaccination, and eight items assessing general attitudes toward the vaccine, both using five-point Likert scales (1 = strongly disagree to 5 = strongly agree). The overall attitude score is the sum of both components (the maximum score is 75, and the minimum is 15). The higher the score of both knowledge and attitude scales, the better the knowledge and the more positive the attitudes.
- **Section 5: **Assessed HPV vaccine uptake, acceptance, intentions, and barriers to uptake (nine items). Higher scores indicated higher levels of knowledge and more positive attitudes.

### Validation of the questionnaire

The questionnaire was reviewed for face and content validity by a panel of three public health experts. Pilot testing of the questionnaire was done on 15 participants, to assess clarity and comprehension of the questionnaire and no modifications needed, the data of pilot testing were not included in the final analysis. Internal consistency was tested by Cronbach’s alpha, which was estimated to be 0.85.

#### Data collection

Data collection was conducted via a Google Form, distributed through all available electronic platforms (e-mail, WhatsApp, Facebook, and Telegram), with the assistance of students’ union leaders who facilitated its dissemination.

#### Data analysis

Data analysis was performed using appropriate statistical tests on SPSS version 25 software (SpssInc, Chicago, ILL Company). Normally distributed continuous variables were expressed as mean ± standard deviation (SD), while categorical variables were presented as numbers and percentages. The Chi-square test was used to assess associations between categorical variables. Independent t-tests were used to compare means between two groups, and one-way ANOVA was applied for comparisons among more than two groups. Multiple linear regression analysis was used to identify predictors of the overall HPV vaccine attitudes, binary logistic regression was used to identify the predictors of participants’ HPV vaccine uptake (yes, no answers), and multivariate logistic regression analysis was used to detect the predictors of intentions of not recommending the HPV vaccine to others (not recommending and recommending the vaccine with not sure category as reference category. Before linear regression analysis, the assumptions of normality, linearity, and homoscedasticity were assessed and found to be satisfied. Multicollinearity was evaluated, and no significant collinearity between the included predictors was detected. Independent variables were added simultaneously to the regression model, based on their significance in univariate analysis and theoretical relevance (enter method). Variables with *p* < 0.05 in univariate analysis were entered into the multivariate model. A p-value ≤ 0.05 was considered statistically significant.

## Results

### Socio-demographic and personal characteristics of the study participants

Approximately 45% of the participants were between 18 and 20 years old. Of the total, 64.2% were females, 93.5% were single, 96.8% were Egyptians, and 64.2% were in the first three faculty grades. Additionally, 2.8% of the participants reported a positive family history of cervical cancer (Table 1).

**Table 1:** Socio-demographic and personal characteristics of the study participants.

Variables (*n*.=601)		*N*	%
Age (years) Minimum-maximum (16–30) Mean ± SD (21.43 ± 2.48)	18–20 21–23 24–26 >26	272 170 152 7	45.3% 28.3% 25.3% 1.2%
Sex	Male Female	215 386	35.8% 64.2%
Residence	Urban Rural	318 283	52.9% 47.1%
Marital status	Single Married	562 39	93.5% 6.5%
Nationality	Egyptian Non Egyptian	582 19	96.8% 3.2%
Academic level	1–3 academic years 4–5 academic years Interns	386 52 163	64.2% 8.7% 27.1%
GPA Minimum-maximum (1.5-6) Mean ± SD (3.46±0.79)	1.50–2.50 2.51–3.50 3.51–4.5 >4.50	18 330 174 7	3% 54.9% 28.9% 1.2%
University	Governmental universities National/private universities	489 112	81.4% 18.6%
University name	Benha University Suez University Kafr El- Sheikh University Mansoura University Cairo University Menoufia University Tanta University Helwan University National Universities Private universities	151 35 40 34 55 91 39 44 60 52	25.1% 5.8% 6.7% 5.7% 9.1% 15.1% 6.5% 7.3% 10% 8.7%
Family history of cervical cancer	Positive Negative	17 584	2.8% 97.2%

#### HPV knowledge

##### General knowledge regarding HPV and its vaccine

31.4% of the students were unaware that HPV is available for both males and females, and 62.1% had no idea that sexually active teenagers shouldn’t get tested for HPV before getting vaccinated. 45.1% of the participants were unaware that the vaccine is not licensed for females over the age of 26. (Table 2).

**Table 2:** Distribution of Participants’ knowledge regarding HPV and its vaccine.

General knowledge regarding HPV and its vaccine(*N* = 601)	Correct answer *N* %	Incorrect answer *N* %	Don’t know *N* %
HPV is a relatively uncommon sexually transmitted infection	345 57.4%	12220.3%	13422.3%
Almost all cervical cancers are caused by HPV infection	27545.8%	16928.1%	15726.1%
The incidence of HPV in women is highest among women in their 30s	31953.1%	82 13.6%	20033.3%
Most people with genital HPV infections are symptomatic	25041.6%	15926.5%	19231.9%
Genital warts are caused by the same HPV types that cause cervical cancer	18430.6%	23639.3%	18130.1%
Sexually active adolescents should be tested for HPV before starting HPV vaccination	8013.3%	37362.1%	14824.6%
The HPV vaccine is not licensed for females older than 26 years of age	149 24.8%	18130.1%	27145.1%
The HPV vaccine is available for both females and males	36260.2%	508.3%	18931.4%
Women and men who have been diagnosed with HPV should not be given the HPV vaccine	174 29.0%	17729.5%	25041.6%

Knowledge of HPV infection among females (*N* = 601)	Agree *N* %	Neutral *N* %	Disagree *N* %
Females are at risk for HPV infection	46777.7%	113 18.8%	213.5%
HPV infection is common among females	41268.6%	15425.6%	355.8%
Genital and anal warts can cause serious physical, emotional, and financial consequences for female	46577.4%	10317.1%	335.5%
Nearly all sexually active females have already been infected with HPV by age 26.	22437.3%	19131.8%	18630.9%
HPV infections may contribute to anal, vulvar, lining of the vagina, cervical and rectal cancers in females	41468.9%	14624.3%	416.8%

##### Knowledge regarding HPV and female vaccination

According to 37.3% of the participants, by the age of 26, nearly all sexually active females have contracted HPV. 68.9% of the participants concurred that HPV infections may be a contributing factor to female malignancies in the anal, vulvar, vaginal lining, cervical, and rectal regions (Table 2).

### Participants’ attitude towards the HPV vaccine

The overall mean attitude score was 44.9 ± 7.6. About half of the participants expressed negative attitudes and concerns regarding the vaccine, citing its perceived novelty, safety concerns, and efficacy concerns (44%, 56%, and 59%, respectively). A total of 28.6% of the participants opposed HPV vaccination due to moral or religious reasons.

Approximately half of the participants (43.4%) were unaware that the vaccine is available for both males and females. For 43.1% of the students, it was easier to administer the HPV vaccine to women than to men.

Half of the participants were concerned about the cost of the vaccine. A total of 28.8% asserted that vaccinating males was unnecessary, as females had already received the vaccination. Additionally, 34.4% believed that HPV causes only a few cancers, and therefore, there was no need to vaccinate females. Furthermore, 44.8% of the participants disagreed with the notion that vaccinating females is illogical since genital and anal warts can be treated through alternative methods (Table 3).

**Table 3:** Distribution of participants’ attitudes toward the HPV vaccine.

Attitude items (*N* = 601) : mean ± SD (44.9 ± 7.6)	Agree *N*. %	Neutral *N*. %	Disagree *N*. %
Worries about the novelty of the vaccine	26443.9%	234 38.9%	10317.1%
Worries about the safety of the HPV vaccine	33,956%	179 29.8%	8313.8
Worries about the efficacy of the HPV vaccines	35,259%	17,329%	7613%
Unaware that the vaccine is available for both males and females	26143.4%	18530.8%	15525.8%
Interested in the HPV vaccine for females	36360.4%	18330.4%	559.2%
The HPV vaccine is more comfortable for females than for males	25943.1%	22036.6	12220.3%
Concerned about the cost of the vaccine	31151.7%	21435.6%	7612.6%
We should vaccinate males against HPV to protect their future partners from cervical cancer and other related diseases	46176.7%	11118.5%	294.8%
Vaccination of females against HPV means that there is no need to vaccinate males as well	17328.8%	16527.5%	26343.8%
It would be important to vaccinate females against HPV to prevent them from getting genital and anal warts	45375.4%	11919.8%	294.8%
HPV causes too few cancers among females to vaccinate them against it	20734.4%	18630.9%	20834.6%
Vaccination of females is unnecessary because genital and anal warts can be managed through alternative methods	14924.8%	18330.4%	26944.8%
It would be important to vaccinate females against HPV to prevent males from getting infected with HPV	38263.6%	17729.5%	427.0%
It is too late to vaccinate against HPV, if an adolescent female is already sexually active	18030.0%	18230.3%	23939.8%

### Relation between participants’ socio-demographic and personal characteristics regarding their knowledge and attitude scores

#### Knowledge score

The overall knowledge score of the participants was statistically significantly different based on their age (*p* = 0.001). Participants aged 21–23 years had significantly higher scores (30.97 ± 6.79) than those in other age groups. The knowledge score was statistically significantly different based on place of residence (*p* = 0.002). Participants from rural areas had significantly higher knowledge scores (30.08 ± 6.87). Furthermore, knowledge scores differed significantly based on academic level, with those in advanced levels (4th and 5th ) demonstrating higher scores. The knowledge scores varied significantly according to GPA (*p* = 0.03), with participants who had a GPA between 3.51 and 4.50 scoring significantly higher (30.20 ± 7.04) than those with lower GPAs. Additionally, there was a statistically significant difference in knowledge score regarding university type, where students from a governmental university had a higher knowledge score compared to those from national and private universities (Table 4).

**Table 4:** Comparison between sociodemographic and personal characteristics regarding overall knowledge, overall attitude, attitude towards HPV vaccine, and attitude towards female vaccination mean scores.

Variables (*N*.=601)	Overall knowledge (Min- Max) (5–41) Mean ± SD	Overall attitude (Min- Max) (15–75) Mean ± SD	Attitude towards HPV vaccine (Min- Max) (8–40) Mean ± SD	Attitude towards female vaccination (Min- Max) (7–35) Mean ± SD
Age (years) 18–20 21–23 24–26 ˃26	27.81 ± 7.78 30.97 ± 6.79 29.26 ± 5.95 30.85 ± 9.87	44.98 ± 7.59 44.22 ± 8.09 46.01 ± 6.73 41.42 ± 12.98	20.96 ± 5.22 20.20 ± 5.37 21.77 ± 4.65 19.42 ± 6.47	24.01 ± 5.36 24.02 ± 5.89 24.23 ± 4.710 22 ± 7.91
ANOVA P-value	0.001**	0.116	0.045	0.755
Gender Male Female	28.49 ± 8.05 29.47 ± 6.68	45.23 ± 8.50 44.84 ± 7.10	20.96 ± 5.48 20.92 ± 4.99	24.26 ± 6.06 23.92 ± 4.98
Students t-test P-value	0.111	0.556	0.931	0.453
Residence Urban Rural	28.26 ± 7.40 30.08 ± 6.87	45.86 ± 7.24 44.20 ± 7.88	21.24 ± 5.14 20.66 ± 5.17	24.62 ± 5.28 23.53 ± 5.44
Students t-test P-value	0.002**	0.008	0.177	0.013
Marital status Single Married	29.07 ± 7.32 29.79 ± 5.26	44.92 ± 7.64 45.84 ± 7.43	20.91 ± 5.12 21.33 ± 5.84	24.01 ± 5.44 24.51 ± 4.65
Students t-test P-value	0.54	0.467	0.622	0.578
Family history of cervical cancer Yes No	30.82 ± 5.91 29.07 ± 7.24	49.35 ± 10.11 44.85 ± 7.51	22.11 ± 5.48 20.90 ± 5.15	27.23 ± 6.04 23.95 ± 5.34
Students t-test P-value	0.32	0.017	0.340	0.013
Academic level 1–3 academic year 4–5 academic year Interns	28.61 ± 7.96 30.59 ± 7.61 29.84 ± 5.66	44.82 ± 7.88 43.48 ± 8.17 45.85 ± 6.72	20.84 ± 5.35 19.53 ± 5.10 21.59 ± 4.62	23.97 ± 5.54 23.94 ± 6.26 24.26 ± 4.70
ANOVA P-value	0.05*	0.114	0.037	0.836
GPA 1.50–2.50 2.51–3.50 3.51–4.50 ˃4.50	27.38 ± 9.9128.61 ± 7.05 30.20 ± 7.04 26.71 ± 9.53	44.16 ± 9.38 45.48 ± 7.45 44.14 ± 7.69 47 ± 8.67	19.22 ± 5.84 21.31 ± 5.20 20.37 ± 5 23 ± 4.69	24.94 ± 7.14 24.17 ± 5.29 23.76 ± 5.33 24 ± 7.702
ANOVA P-value	0.03*	0.179	0.055	0.737
Governmental universities National / private universities	29.72 ± 6.78 26.50 ± 8.39	45.23 ± 7.18 43.91 ± 9.28	21.03 ± 4.96 20.50 ± 5.98	24.19 ± 5.26 23.41 ± 5.91
Students t-test P-value	0.0001	0.098	0.320	0.166

*Significant difference (*p* ≤ 05) & highly Significant difference (*p*≤0.01) * *.

#### Attitude scores

This study revealed a statistically significant difference in the overall attitude level of participants based on their residency type and family history (p values = 0.008 and 0.017, respectively). The data showed that individuals from rural areas had a higher mean of overall attitude (45.86 ± 7.24) compared to urban individuals (44.20 ± 7.88). Also, those with positive family history have a higher mean overall attitude score (49.35 ± 10.11) compared to those with negative family history (44.85 ± 7.51). The data on attitudes toward the HPV vaccine indicated statistically significant differences regarding age groups and academic levels (p value = 0.045 & 0.037, respectively) where the highest mean scores were reported in the 18–20 age group (20.96 ± 5.22) and among interns’ participants (21.59 ± 4.62). For attitudes towards female vaccination: there were statistically significant differences among participants based on their residency type and family history status (p value = 0.013 & 0.013 respectively), with higher means of attitude among those from rural areas (24.62 ± 5.28) and individuals with positive family history (27.23 ± 6.04).

### sources of knowledge regarding HPV and its vaccine

**Fig. 1:**
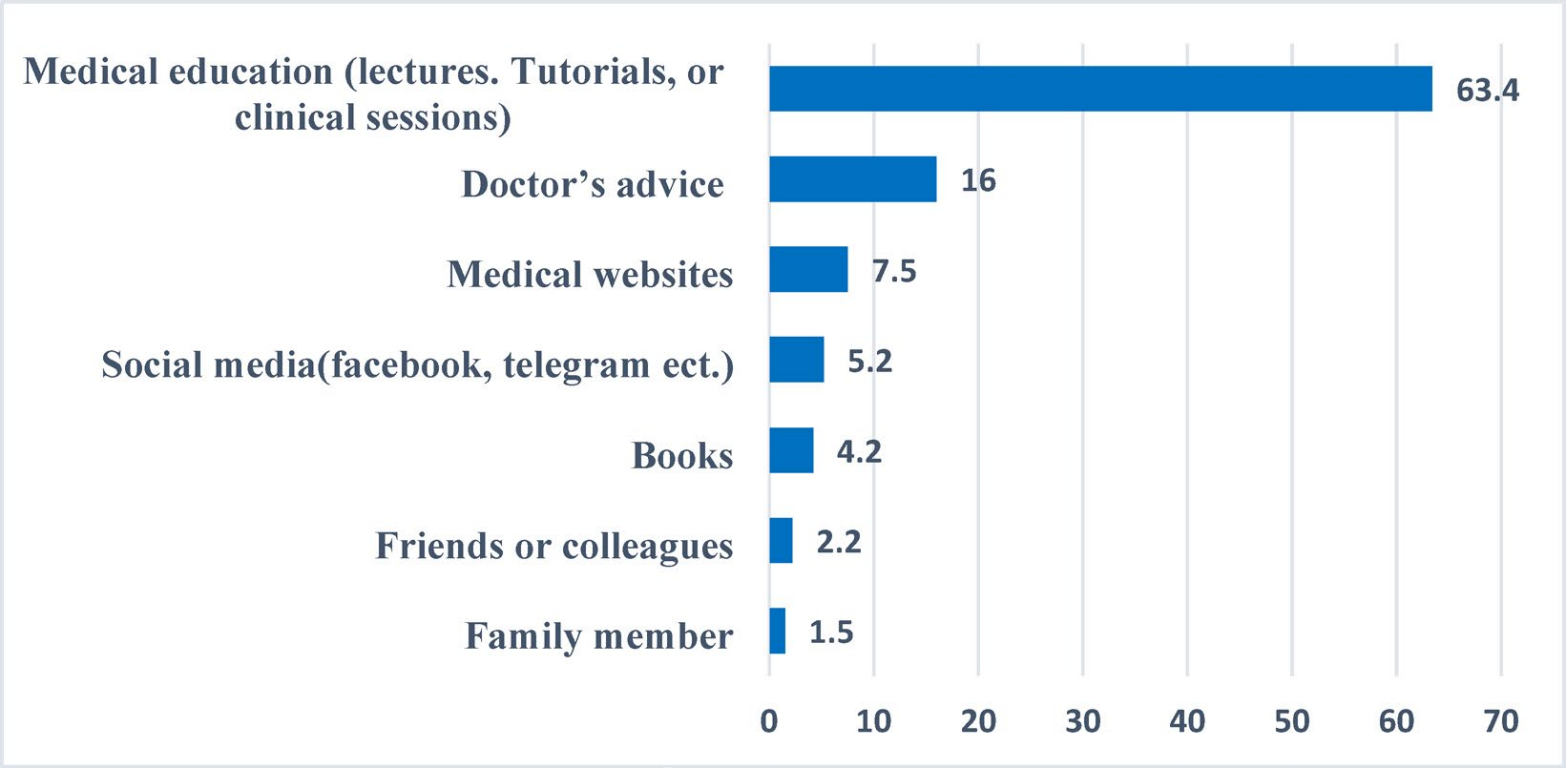
Distribution of main sources of knowledge regarding the HPV vaccine.

For 63.5% of the participants, the HPV vaccine was primarily understood through medical education, followed by advice from doctors (16%) and medical websites (7.5%) (Fig. 1).

### Acceptance, intentions towards the HPV vaccine, and gender differences among the studied participants

The HPV vaccine had not been taken by 87.9% of the participants, while 42.6% were inclined to take it, and 44.3% were hesitant to do so. 58.9% of the participants expressed willingness to receive the HPV vaccine if available for free, and 57.9% indicated their intention to recommend it to others.

This study revealed significant gender differences in vaccine uptake (*p* = 0.001). A higher percentage of females (91.2%) had not received any dose of the HPV vaccine compared to males (81.9%), highlighting a substantial gender disparity in vaccine acceptance or access. The distribution across different dose categories revealed that a small number of females had received one dose (3.9%), three doses (3.1%), with smaller percentages having received two doses (1.8%).

Furthermore, the study revealed differences in willingness to recommend the HPV vaccine between males and females. The data showed a significant difference (*p* = 0.009), with a higher percentage among females (59.3%) willing to recommend the vaccine compared to males (55.3%) (Table 5).

**Table 5:** Acceptance, future intentions towards the HPV vaccine and gender differences among the studied participants.

Uptake and intentions towards the HPV vaccine	Total (*N* = 601) *N* %		Male (*N* = 215)	Female(*N* = 386)	*P*- value
HPV vaccine uptake No Yes 73 (12.1%) Yes, 1 dose Yes, 2 doses Yes, 3 doses	528 30 23 20	87.9% 5% 3.8% 3.3%	176 81.9% 8 3.7% 13 6.0% 18 8.4%	352 91.2% 15 3.9% 7 1.8% 12 3.1%	0.001^**^
Willingness to take the HPV vaccine (N= 528) No Hesitant Yes	69 234 225	13.1% 44.3% 42.6%	33 15.3% 82 38.1% 100 46.5%	45 11.7% 171 44.3% 170 44.0%	0.140
Willingness to take the HPV vaccine if it is free (N= 528) No Hesitant Yes	59 158 311	11.2% 29.9% 58.9%	33 15.3% 60 27.9% 122 56.7%	38 9.8% 122 31.6% 226 58.5%	0.120
Willingness to recommend HPV to others Yes No Hesitant	348 55 198	57.9% 9.2% 32.9%	30 14.0% 66 30.7% 119 55.3%	25 6.5% 132 34.2% 229 59.3%	0.009^**^
Opposition to HPV vaccination for moral or religious reasons Yes No Not sure	172 240 189	28.6% 39.9% 31.4%	69 32.1% 80 37.2% 66 30.7%	102 26.4% 159 41.2% 125 32.4%	0.327

### Reasons for taking and recommending the HPV vaccine to others

**Fig. 2:**
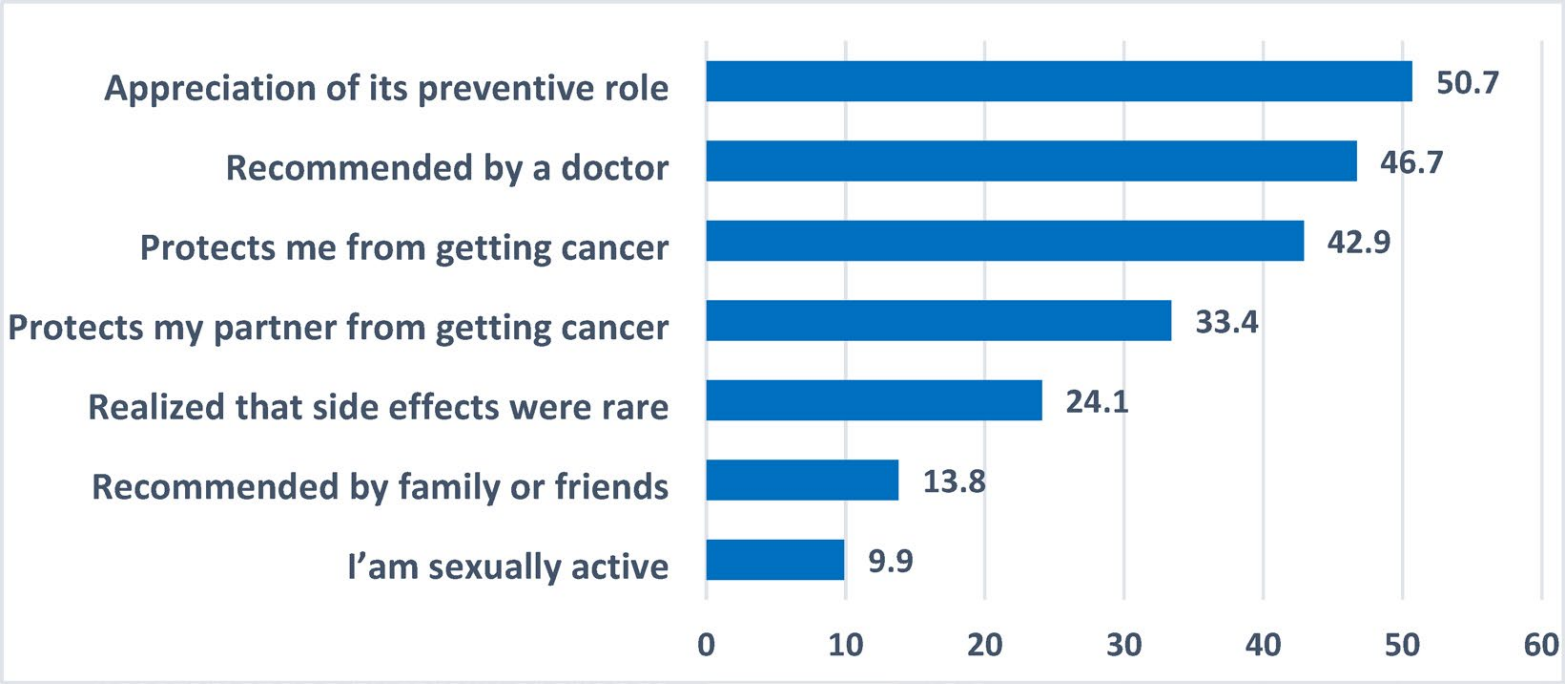
Reasons for taking and recommending the HPV vaccine to others. N.B. More than one answer was allowed.

Half of the participants (50.7%) would recommend the HPV vaccine to others because they value its preventive role against cervical cancer. A total of 46.7% would suggest it based on a doctor’s recommendation, and 42.9% to protect others from cervical cancer, while 9.9% would recommend it due to their sexual activity (Fig. 2).

### Predictors of participants’ attitude toward the HPV vaccine

The participants’ age, residence, and familial history of cervical cancer significantly predicted their attitudes toward the HPV vaccine and total knowledge score regarding HPV and its vaccine (B [95% CI] = 1.53 [1.37–1.69] (*p* = 0.000), 2.23 [0.90–3.65] (*p* = 0.002), 7.13 [2.76–11.49] (*p* = 0.001), and 0.27 [0.17–0.36] (*p* = 0.000), respectively) (Table 6).

**Table 6:** Regression models for the predictors of participants’ attitudes toward the HPV vaccine, HPV vaccine uptake, and intentions of not recommending the HPV vaccine to others.

Multiple linear regression model for the predictors of participants’ attitudes toward the HPV vaccine.		Total attitude score	
		B (95% CI)	*P*-value
Age		1.53 (1.37–1.69)	0.000* *
Residence (ref: rural)		2.23 (0.90-3.65)	0.002* *
Family history ( ref: no)		7.13 (2.76–11.49)	0.001* *
Total knowledge score		0.27 (0.17 − 0.36)	0.000* *

Binary logistic regression model for the prediction of participants’ HPV vaccine uptake		Adjusted OR (95% CI)	P-value
Family history: Yes (No: reference)		8.09 (2.96–22.09)	0.000* *
Total knowledge score		0.94 (0.90 − 1.00)	0.05*
Total attitude score		0.97 (0.95-0.99)	0.002* *

Multivariate logistic regression for the predictors of intentions of not recommending the HPV vaccine to others		Adjusted OR (95% CI)	P-value
Not -recommending the vaccine	Gender: male (ref: female)	2.740(1.48–5.04)	0.001**
	Marital status: single (ref: married)	0.318 (0.123- 0.826)	0.019*
Recommending the vaccine	Gender: male (ref: female)	0.933(0.642-1.355)	0.716
	Marital status: single (ref: married)	1.519(0.683 − 3.380)	0.306

Reference category in multivariate logistic regression is (not sure).

*Significant difference (p ≤ 05) & highly Significant difference (p ≤0.01) * *.

### Predictors of participants’ uptake of the HPV vaccine

The adoption of HPV vaccines was significantly predicted by the participants’ overall attitude toward HPV vaccines and their familial history of cervical cancer (adjusted OR [95% CI] = 8.09 [2.96–22.09] (*p* = 0.000) and 0.97 [0.95–0.99], respectively). Additionally, the participants’ adoption of the HPV vaccine was significantly predicted by their total knowledge score of HPV and its vaccine (*p* < 0.05) (Table 6).

### The predictors of not recommending the HPV vaccine to others

Multivariate logistic regression for identifying significant predictors influencing the likelihood of not recommending the HPV vaccine to others indicates that gender, and marital status are significant predictors, where males are significantly more likely to not recommend the vaccine compared to females (Adjusted OR = 2.740, *p* = 0.001). Also, single individuals are less likely not to recommend the vaccine compared to married individuals (Adjusted OR = 0.318, *p* = 0.019) (Table 6).

### Barriers to the HPV vaccine uptake among the participants

**Fig. 3:**
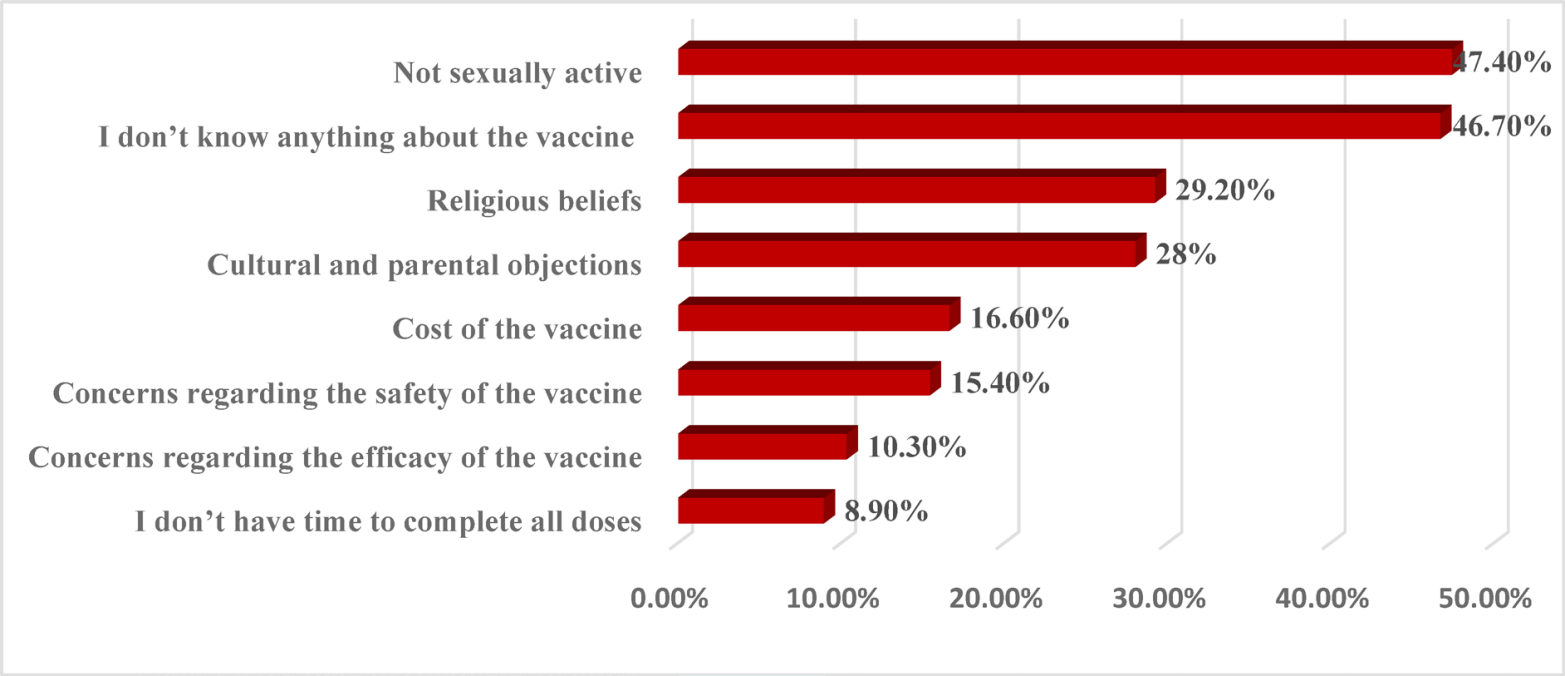
Barriers to HPV vaccine uptake among the participants. N.B. More than one answer was allowed.

On exploring the barriers towards HPV vaccination, the results revealed that 47.4% of the participants didn’t take the vaccine because they were not sexually active, and 46.7% because they didn’t know anything about the HPV vaccine. Surprisingly, about one-third (28%) of the participants didn’t take the vaccine because of cultural and parental objections, and 29.20% perceived themselves as not susceptible to HPV infection, as they are religious. A total of 34.4% of the participants did not receive the HPV vaccine because they were unsure where to obtain it. The high cost of the vaccine was cited as a barrier by 16.6% of the participants **(**Fig. 3**).**

## Discussion

Persistent HPV infection of the cervix is responsible for 95% of cervical cancers if treatment is not administered^3^. In addition to exploring the variables that impact the acceptability and adoption of the HPV vaccine, this cross-sectional study aimed to assess the attitudes and knowledge of medical university students regarding HPV and its vaccine. The findings indicate that most participants were young medical students, predominantly female and single, reflecting the typical demographic composition of university medical programs in Egypt. Understanding the perceptions of this group is particularly important, as future healthcare professionals play a critical role in promoting HPV awareness, addressing misconceptions, and encouraging vaccine uptake among the wider population.

### Knowledge regarding HPV and its vaccine

In the current study, HPV was identified as a prevalent sexually transmitted infection by 57.4% of respondents, while 45.8% believed that it is responsible for the majority of cervical cancers. This proportion is higher than that reported by Ghazy et al. 2024^16^, who found that only 30% of participants were aware that HPV is a sexually transmitted disease and considered it a significant cause of cervical cancers.

The study found that a significant portion of the participants were unaware of two points: first, that genital HPV can be asymptomatic, and second, that the types of HPV causing genital warts differ from those causing cervical cancer. Our findings are consistent with those of previous research^17^. Similarly, a study conducted in Saudi Arabia found that 60.2% of participants were aware that the vaccine is available for both genders^15^. This study revealed that 45.1% of the participants were unaware that the HPV vaccine is not licensed for females over the age of 26. Similarly, this was reported by Darraj et al., 2022^14^. Conversely, 71.7% of participants correctly responded that the HPV vaccine is not prescribed for women over the age of 30 in a study conducted in Turkey by Kayı et al., 2020^18^.

Knowledge gaps among the studied medical students may be arising from deficiencies in the academic education system, particularly its limited integration of theoretical knowledge with practical applications in different situations and real-life practice.

#### Sources of knowledge regarding HPV and its vaccine

The results of this study indicated that medical education was the primary source of HPV vaccine knowledge for 63.5% of participants, followed by clinician advice (16%) and medical websites (7.5%). Similarly, according to Sallam et al., 2021, university courses were the primary source of HPV knowledge for 65.1% of students^19^. Trusted sources, such as healthcare professionals and educators, primarily provide credible information. In contrast, Khatiwada et al., 2021 found that media sources, such as radio, television, and the internet, were the primary channels of HPV vaccine information for students in Indonesia, highlighting the potential of media in effectively spreading awareness^20^.

### Attitude towards HPV vaccines

The current study demonstrated that approximately half of the participants had negative attitudes and concerns regarding the novelty, safety, and efficacy of vaccines. These concerns reflect a degree of vaccine hesitancy even among individuals with medical backgrounds, which may negatively influence future vaccine advocacy and public health promotion. Similarly, in 2023, a study by Abdelaliem et al. conducted in Saudi Arabia revealed that 55.4% of respondents believed the vaccine was novel, and 49% were concerned about its efficacy and safety^15^. Such concerns may arise from limited exposure to detailed information about HPV vaccination or misconceptions regarding vaccine development and safety monitoring.

Based on the findings, financial considerations emerged as an important barrier to vaccine acceptance, as many participants reported concerns regarding the cost of vaccination. This may be explained by the fact that they are students and often primary financial dependents within their families. A study conducted in Saudi Arabia by Abdelaliem et al. found that 43% of participants were concerned about the vaccine’s cost, which is lower than the rate reported in the current study^15^. Besides, the price was a concern for 36% of respondents, as indicated by the data in Darraj et al., 2022^14^. These findings highlight the potential influence of economic factors on vaccine acceptance and suggest that financial barriers may limit HPV vaccine uptake in countries where the vaccine is not included in national immunization programs.

### Participants’ socio-demographic and personal characteristics regarding their knowledge and attitude scores

The present study’s findings demonstrated a statistically significant difference in the knowledge scores of the participants according to their age (*p* = 0.001). This outcome can be attributed to the students’ growth and development during adolescence and adulthood, as they become more interested in women’s health awareness, in addition to the accumulation of academic medical knowledge over time. The results of this research are consistent with a Saudi study by Abdelaliem et al. which demonstrated a significant relationship between the age of nursing students and their knowledge of HPV^15^. In contrast, a study conducted by Darraj et al., 2022 found no significant correlation between the age of the participants and their knowledge of HPV^14^. These inconsistencies across studies may be related to differences in educational curricula, levels of clinical exposure, and variations in public awareness of HPV across different settings.

The current study revealed a statistically significant difference in the participants’ attitude scores, with those having a family history of cervical cancer exhibiting significantly higher scores than those without such a history. Similarly, the findings of Abdelaliem et al.^15^. This could be explained by the fact that individuals with a personal connection to affected cases may be more motivated to learn about the disease as a protective measure. Additionally, witnessing the challenges faced by a family member with cervical cancer may create an emotional drive to prevent similar experiences in themselves or their loved ones, fostering a proactive and positive attitude toward the HPV vaccine.

### HPV vaccine uptake

This study reported that 87.9% of participants had not received the HPV vaccine highlighting a critical gap between awareness and actual vaccination uptake. Similarly, a study carried out in Beirut by Hourani et al. in 2024 reported that the majority of participants (71.1%) had never received the HPV vaccine^17^. Additionally, a study conducted in Egypt by Fouda & Abdelsalam in 2024 reported that only 2% had received the HPV vaccine^21^. The extremely low percentage of participants who received the HPV vaccine can be attributed to a lack of awareness and knowledge regarding the vaccine and its benefits, as highlighted by the significant proportion of individuals who were unaware of its availability. Furthermore, the cost of the vaccine, which is not subsidized in Egypt and is not included in the national immunization program, may contribute to its low uptake.

### Intentions towards HPV vaccines

This study showed that 42.6% of participants were willing to take the HPV vaccine, while 44.3% were hesitant. Similarly, a study conducted by Li et al., 2024 in China^22^. The lower willingness observed among Egyptian students may reflect limited knowledge and awareness of HPV and its vaccine, highlighting persistent gaps in education and public health messaging. Limited awareness campaigns and educational efforts may prevent participants from fully understanding the benefits of the vaccine. Additionally, the cost of the vaccine and its availability only at VACSERA sites may hinder access.

The present study found that approximately 57.9% of participants were willing to receive the HPV vaccine if provided at no cost. A study conducted by Zakzook et al., 2022 at Mansoura University’s faculties revealed that 76.3% of participants were willing to receive the vaccine for free, while only 32.9% were willing to pay for vaccination^13^. These findings underscore the critical role of cost as a determinant of HPV vaccine uptake, particularly in contexts where the vaccine is not included in national immunization programs.

For moral or religious reasons, 28.6% of participants in this study opposed HPV vaccination, which contrasts with Abdelaliem et al., 2023, where approximately half of the participants shared the same opposition for the same reasons^15^, while, Redd et al., 2022 found that religious practices have a positive impact on attitudes and knowledge regarding HPV vaccines, suggesting that religious observance may serve as a protective factor against HPV infection^23^. Taken together, these findings highlight the complex interplay of economic, cultural, and religious factors in shaping vaccine acceptance, emphasizing the need for culturally sensitive educational interventions alongside accessible vaccination programs.

### Predictors of attitudes towards HPV vaccines

This study revealed that a family history of cervical cancer was a significant predictor of attitudes toward the HPV vaccine (OR = 7.13). Students who had a family history of cervical cancer exhibited a more favorable perspective toward HPV and its vaccine than those who did not. This can be attributed to the fact that students with a family history of cervical cancer are more inclined to research the disease and acquire a greater understanding and awareness of it. This result was supported by Darraj et al., 2022 and Oh et al., 2023^14,24^.

The overall knowledge score of HPV and its vaccine was another major indicator of attitude toward the HPV vaccine. This finding is consistent with those of Abdelaliem et al.,2023 and D’Errico et al.,2021, who reported that participants with a more thorough understanding of HPV were more likely to view HPV and its vaccination favorably^15,25^. In addition, age was a significant predictor of participants’ attitudes toward the HPV vaccine, which may be influenced by the source of information, typically improving their general attitude.

### Predictors of HPV vaccine uptake

Moreover, family history of cervical cancer was identified as a significant predictor of HPV vaccine uptake, aligning with studies by Rezqalla et al. and He et al. showing that family history increases HPV-related knowledge and vaccination uptake^26,27^. Positive attitudes toward the HPV vaccine significantly influenced uptake, as students with positive attitudes were more likely to receive the vaccination, supported by systematic reviews and meta-analyses by Agimas et al. and Addisu et al.^28,29^.

Additionally, total knowledge scores about HPV and its vaccine had a positive impact on vaccine uptake, consistent with findings from studies in Italy and China by Cocchio et al. and You et al.^30,31^. This highlights the significance of knowledge and attitudes in fostering evidence-based decisions and promoting preventive health practices.

Despite the fact that 47.4% of the participants in this study thought they were safe since they did not engage in sexual activity, new molecular-epidemiological research indicates that HPV may also spread through non-sexual routes such as contaminated surfaces^32^. Another important route is vertical transmission. According to 2024 cohort research by Nantel et al., infants born vaginally had a considerably higher incidence of HPV detection than those born via caesarean section, indicating that perinatal transmission is a serious danger^33^. These findings challenge the misconception that sexual inactivity ensures complete protection and emphasize the broader protective value of HPV vaccination, which safeguards against both sexually and non-sexually acquired infections. This reinforces the need for educational interventions that clarify HPV transmission routes; ensuring students understand the importance of vaccination irrespective of perceived sexual risk.

### Barriers to the HPV vaccine uptake among the participants

Cultural and parental influences emerged as important barriers to HPV vaccination in the present study. Approximately 28% of participants reported not receiving the vaccine due to cultural norms or parental objections, reflecting the role of societal values in shaping vaccination behaviour. This finding aligns with a systematic review by Salleh et al. (2025), which identified moral values and reluctance to discuss sexual topics as key determinants of HPV vaccine refusal^34^. Additionally, 29.2% of participants believed that their religious beliefs protected them from HPV infection. While religiosity may influence perceptions of risk, evidence suggests that vaccine refusal is not always directly predicted by religious adherence. For example, a 2024 study of Black and Hispanic parents reported conflicting relationships between religious attendance, salience, and vaccination intention, indicating that faith-sensitive educational interventions could effectively address misconceptions^35^.

### The predictors of not recommending the HPV vaccine to others

The study also identified key predictors of not recommending the HPV vaccine to others. Being male and having lower knowledge levels were significantly associated with a reduced likelihood of vaccine recommendation. These findings are consistent with Wang (2024), who found that lower HPV-related knowledge among young Chinese men was strongly linked to decreased support for women’s vaccination^36^. Similarly, Taha et al. (2025) reported that male medical students in Jordan were less likely to recommend vaccination and to self-vaccinate^37^. Together, these results highlight the critical importance of addressing gender differences and knowledge gaps when designing educational interventions to improve HPV vaccine acceptance and advocacy among future healthcare professionals.

## Study strengths and limitations

This study has some limitations. First, the use of convenience and snowball sampling may introduce selection bias, which is represented by the higher percentage of junior students’ participation as it depended on voluntary response and access to online platforms. Second, the data were self-reported, which may be subject to recall bias and social desirability bias, potentially affecting the accuracy of responses. Third, response bias may have occurred if students with greater interest in HPV-related topics were more likely to participate in the survey. Finally, the cross-sectional design of the study limits the ability to establish causal relationships between knowledge, attitudes, and HPV vaccine uptake.

Despite these limitations, the study included a relatively large and diverse sample of medical students from multiple universities in Egypt, which enhances the robustness and relevance of the findings.

## Conclusions and recommendations

According to this study’s findings, despite being medical students, they demonstrated gaps in knowledge regarding HPV and its vaccines. Notably, the majority of the students had not received the vaccine, while about half of them were inclined to take it or were hesitant. Additionally, about two-thirds of the participants expressed a willingness to receive the HPV vaccine if it were provided for free. Exploration of barriers to vaccination revealed that one-third didn’t take the vaccine due to religious beliefs or due to cultural and parental objections.

Negative attitudes regarding certain vaccine-related issues suggest gaps in knowledge and attitudes among medical students, which may reflect broader knowledge gaps within the community that highlight the need for:

- Well-designed, systematic HPV education that focuses on targeted age groups and their parents as their guardians and families through all channels.
- Integration into various medical curricula to provide comprehensive knowledge capable of changing misconceptions and negative attitudes, as the current medical students will be the future health professionals and parents, and will play a crucial role in mobilizing the public towards the necessity of vaccine uptake.
- National health authorities and planners should allocate resources and efforts to make the HPV vaccine available, accessible and affordable.

## Data Availability

All data that support the research finding are available from the corresponding author upon request from the editor.

## Abbreviations

HPV: Human Papilloma Virus
WHO: World Health Organisation
STDs: Sexually Transmitted Diseases
